# *Heyndrickxia coagulans* SANK70258 supplementation improves growth performance, gut health, and liver function in growing pigs

**DOI:** 10.3389/fvets.2025.1537913

**Published:** 2025-05-27

**Authors:** Masanori Aida, Ryouichi Yamada, Takahiro Kawase, Toshiki Matsuo, Takamitsu Tsukahara

**Affiliations:** ^1^Science and Innovation Center, Mitsubishi Chemical Corporation, Yokohama, Japan; ^2^Kyoto Institute of Nutrition and Pathology, Kyoto, Japan; ^3^Mitsubishi Chemical Corporation, Tokyo, Japan

**Keywords:** growing pig, growth performance, leaky gut, liver function, probiotics, *Heyndrickxia coagulans* SANK70258

## Abstract

*Heyndrickxia coagulans* is a Gram-positive, facultatively anaerobic, and spore forming bacterium. This species is often used as probiotics, therefore it is known for its health-conferring effects on livestock and humans. Previously, we showed that administering *H. coagulans* SANK70258 (SANK70258) to coccidiosis-infested broilers improved their growth performance by inducing anti-inflammation. Nonetheless, a few studies have observed the effects not only of *H. coagulans* spp. but also of SANK70258 in pigs. Here, we evaluated if SANK70258 could help improve the growth of pigs from weaning (days 0–42), growing (days 42–84) to fattening (days 84–126) periods. Twenty-four weaned crossbred (Duroc × Large White × Landrace) piglets were divided into control (CC; 4 replicants, *n* = 8) and 0.01% SANK70258 supplementation (P; 8 replicants, *n* = 16) groups. Diets and water were given *ad libitum*. After the weaning period (day 42), the pigs in group P were further sub-divided into pens with (PP; 4 replicants, *n* = 8) or without SANK70258 administration (PC; 4 replicants, *n* = 8). SANK70258 improved growth performance during the growing period [statistical differences were observed on days 42–56 (PP) and 70–84 (PP and PC)]. During weaning, a period well recognized as a frequent pathogen infection stage, due to the portal blood of pigs with leaky guts flows into the liver carrying gut microbes and their products such as endotoxin and bacterial DNA. P pigs experienced an improved liver function evidenced by the plasma alanine aminotransferase reduction and hence, a putative improved leaky gut condition evidenced by the plasma lactulose/mannitol ratio reduction. Nonetheless, during the fattening period, the positive effects became confounding with a pathogenic infection expressing a drastic increase in plasma aspartate aminotransferase on day 126. Nonetheless, stearate composition in meat of PP pigs were observed on day 126. Our results suggested that the SANK70258 administration may be a good natural product to improve the growth performance of pigs during production stages with a lower probability of infection like the growing period.

## 1 Introduction

Pork is one of the most consumed meat globally (1). Indeed, in 2022 alone the consumption of pork in the world reached 113 million tons (2). Thus, growth performance is one of the most important productive traits in pig production (3). However, during weaning, piglets are vulnerable to several conditions that decrease their growth performance. For example, changes in the environment, diet, management and separation from dams exert negative effects on the growth performance of piglets (4). These stressors lead to negative changes in the intestinal health of piglets, which in turn cause diarrhea, slow growth and a decreased ability to withstand diseases (4).

The health of the intestine of the pig is very important to achieve optimal growth, as it is the place where digestion and absorption of nutrients take place (5). In addition, a healthy intestinal barrier protects the piglet from feed-derived antigens, pathogenic microorganisms and other harmful metabolites (5). The intestine of the pig is populated by a diverse microorganism community (6). Indeed, bacterial colonization of the pig’s colon is known to be about 10^10^–10^11^ per g or mL of content (7). The condition of the gut microbiota is linked to the health and growth performance of pigs (8). Apart from the microbial barrier, the intestine of the pig is protected by the mucosal and the immunological barriers (9). A leaky gut occurs when the intestinal barrier is compromised, and intestinal inflammation takes place (10). The portal blood of pigs with leaky guts flows into the liver carrying gut microbes and their products such as endotoxin and bacterial DNA (11). This condition contributes to persistent chronic inflammation and progressive liver damage (12, 13). Probiotics help restore the gut barrier integrity and modulate the microbiota, thus reducing lipopolysaccharide translocation and systemic inflammation (14). We have also described the cell preparation of *Enterococcus faecalis* EC-12, which stimulates the hepatic superoxide disumutase activity (15). Consecutive administration of this strain improves the liver damage such as fatty degradation in murine aged model (16). Thus, probiotics offer a therapeutic avenue for improving liver health. Furthermore, high levels of aspartate aminotransferase (AST) and/or alanine aminotransferase (ALT) in the blood indicate damage of the liver function (17), but a probiotic administration often suppresses these parameters (18).

Probiotics is a generic term to refer to those microorganisms that confer beneficial effects to the host, following ingestion. *Heyndrickxia coagulans*, also known as *Lactobacillus sporogenes* (19) or *Bacillus coagulans* (20) has been shown to modulate the microbiotas and immunostimulate the microbiotas of broilers (21). It is known that some strains of *H. coagulans* have antimicrobial activity (22), and that *H. coagulans* SANK70258 stimulates antimicrobial peptide secretion in the mucosa (23). Gut microbiota modulation by *H. coagulans* SANK70258 administration was at least partly caused by these functions. In addition, *H. coagulans* administration has been shown to be beneficial to patients with alcoholic fatty liver disease (24). An immune modulation effect of *H. coagulans* was also known as the common function (24); some strains of *H. coagulans* such as MTCC5856 (25) and BC198 (26) suppressed the inflammation and/or anti-inflammatory cytokine stimulation. Also, *H. coagulans* SANK70258 can modulate the immune function with suppression of inflammation, as described elsewhere (27). In pigs, several studies have reported the benefits of probiotics to weaning pigs (28, 29). Benefits of these probiotic administration were expected in the form of prevention of pathogen infection (30, 31), because pigs after weaning can be easily infected by many pathogens (32, 33). On the other hand, after the weaning period, the enhancement of growth performance became unclear (34), because pigs no longer need enhanced supplementation to prevent pathogen infections (33). Nonetheless, chronic inflammation, caused by stress and inflammation-inducing ingredients in feed, is always found in intensive livestock production systems; thus anti-inflammation inducing-materials are beneficial to promote growth performance (35). To the best in our knowledge, only one study has described the use of *H. coagulans* for pigs in the growing and fattening periods (36), but the reported growth performance was not improved remarkably. Based on this background, we hypothesized that *H. coagulans* SANK70258, known as an anti-inflammation-inducing probiotic, can enhance the growth performance particularly in pigs during the growing and fattening periods.

The aim of the present study was to assess if the administration of probiotic *H. coagulans* SANK70258 could help improve the growth performance of pigs from the weaning to the fattening periods. Because *H. coagulans* SANK70258 stimulated gene expressions of tight junction protein, suppressed gene expressions of inflammatory cytokines, and modulated the microbiota composition in the gut of broilers (27), we designed three analyses to determine the improvement mechanisms. (1) Plasma AST and ALT concentrations were measured every 2 weeks to evaluate the liver condition. (2) Lactulose/mannitol (L/M) test were observed at the weaning period (day 28) to evaluate the leaky gut condition. (3) mRNA expressions associated with antimicrobial peptide, tight junction proteins, IgA secretion and cytokines in jejunal, ileal, and cecal mucosae were observed. Furthermore, the organic acid concentration in the cecal content, and plasma in the ileal and cecal veins, the cecal microbiome and the fatty acid composition in muscle were also determined.

## 2 Materials and methods

### 2.1 Probiotics

Probiotic *H. coagulans* SANK70258 (SANK70258) manufactured by Mitsubishi Chemical Cooperation (Tokyo, Japan) containing 1 × 10^9^ CFU per g was used. Supplementation was given to groups at 0.01% (w/w), so that diets contained 1 × 10^5^ CFU per g of feed (0.1 g/kg of feed). Based on results from a previous chicken study (21), we expected that this concentration was enough to enhance the growth performance of pigs.

### 2.2 Animals and diet

**Figure 1:**
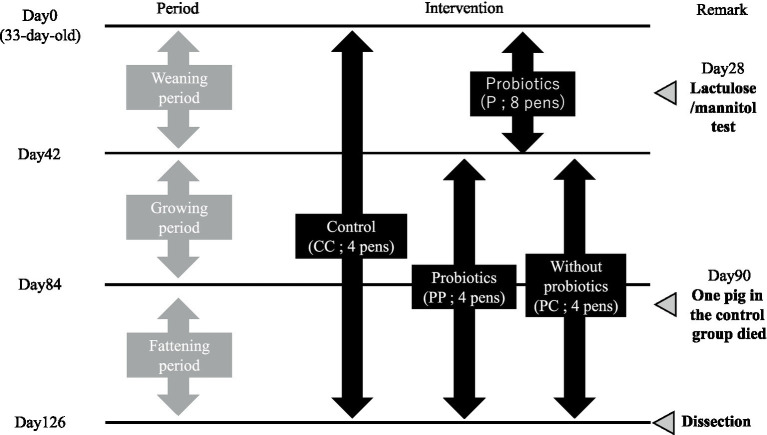
Schematic design of this study. Each pen housed two pigs. However, one pig in C group died on day 90. On day 28, a Lactulose/Mannitol test was performed, and blood was collected from the jugular vein. At dissection on day 126, muscle, blood, cecal contents, and intestinal tissues were sampled. *H. coagulans* SANK 70258 was supplemented to the diet at 1 × 10⁵ CFU/g feed. For statistical analysis, a two-group comparison was conducted during the weaning period, while a multiple comparison test was performed among three groups during the growing and fattening periods.

The experimental design of this study is shown in Figure 1. Twenty-four weaned piglets (Duroc × Large White × Landrace; 30-day-old; 12 castrated male and 12 female) were purchased from a commercial supplier (Oita, Japan) and kept in an open shed at the KYODOKEN Institute (Kyoto, Japan). They were kept in 12 individual pens (W: 1.7 × D: 4.0 m; one castrated male and one female) and acclimatized for 3 days. After acclimatization, pigs in the pens were divided into treatment-free control (CC, 4 pens) and 0.01% SANK70258 supplementation (P, 8 pens) groups. The mean body weight of the pigs in groups was the same (8.8 ± 0.8 kg).

Piglets in each group were housed in a floored-pen with sawdust bedding and equipped with a gas brooder (Nakajima Seisakusyo, Nagano, Japan) to maintain an adequate, stable environment. Feed and drinking water were supplied *ad libitum*. The basal diet was formulated by Marubeni Nisshin Feed Co. Ltd. (Tochigi, Japan). The composition and the nutrient content (%) of the diets are shown in the Supplementary Table S1. The diets were given to the piglets throughout the present study (18 weeks). As the piglets developed, the diets were changed from the weaning period to the growing period on day 42, and then from the growing period to the fattening period on day 84. After the weaning period (day 42), the pens of group P were further sub-divided into SANK70258 administration (PP; four pens) and without SANK70258 administration (PC; four pens) groups. The mean body weight of each group was similar (PP 39.1 ± 1.5 kg vs. PC 37.3 ± 1.9 kg).

### 2.3 Body weight, feed intake measurements and clinical observation

Clinical symptoms such as vitality, appetite, respiratory difficulties and diarrhea were checked individually and the worse score between the two pigs were recorded as the daily score in each pen. The scoring criteria of clinical symptoms were described elsewhere (37). A female pig in CC group had a severe clinical sign of respiratory difficulties in the morning of day 90. A veterinarian checked the symptoms of the animal and injected her Marbofloxacin (Marbocyl 10%, Meiji Animal Health, Tokyo, Japan) and Flunixin meglumine (Forvet 50, MSD Animal Health, Tokyo, Japan). Unfortunately, this pig died in the evening of day 90. The pig was dissected to assess the cause of death. Typical adhesion was observed between the pleura and the lungs. Therefore, a veterinarian diagnosed that the pig died of acute pleuropneumonia. Afterwards, the growth performance of this pen was calculated by the parameters of the remaining castrated male. No other pigs used in the present study showed clinical signs of respiratory difficulties throughout this study. Therefore, the remaining pigs were not treated with antimicrobials or other drugs.

All animals used in the present study were identified with ear tags (odd numbers, male; even numbers, female), and individually weighed on days 0, 14, 28, 42, 56, 70, 84, 98, 112, and 126. In addition, feed intake in each pen was calculated on the same days. Individual daily weight gain (DG) and daily feed intake in pens were calculated, and feed conversion ratio (FCR) in pens were calculated with the two parameters using the following equation: (FCR) = (Daily feed intake in a pen)/(total DG of two pigs in a pen). Separately, blood was collected individually when the body weights were measured. Blood was collected from the jugular vein and immediately cooled on ice. Plasma was separated by centrifugation (3,000 × *g*, 10 min, 4°C; CF9RX, HITACHI, Ibaraki, Japan) and stored at −80°C (VT-208; Nihon Freezer, Tokyo, Japan) until further use.

### 2.4 Slaughter procedure

On days 126 and 127, all pigs were slaughtered. For technical limitations, pigs in pens 1 and 2 were slaughtered on day 126, and the remaining pigs in pens 3 and 4 were slaughtered on day 127. All pigs were injected Mafoprazine mesylate (0.5 mg/kg; Mafropan 1% injection, Bussan Animal Health, Osaka, Japan) and Midazolam (1.0 mg/kg; Dorumicum Injection 10 mg, Maruishi Pharmaceuticals, Osaka, Japan) intramuscularly for sedation. When the pigs were sedated, pentobarbital sodium (12.96 mg/kg; Somnopentyl, Kyoritsu, Tokyo, Japan) was injected intravenously. While under deep anesthesia, the bodies of pigs were midline incised, and blood was collected from the cecal and ileal veins. Then, after exsanguination, the intestines were resected and separated into the small and large intestines. The small intestines were equally cut into eight segments as previously described (38). Segments 2 and 8 were defined as jejunum and ileum, respectively. Digestas in ilea and ceca were collected under sterile conditions, and then immediately cooled on ice. The mucosae of jejuna, ilea and ceca were collected with clean sterile glass slides. The mucosae were soaked into RNA-*later* solution (Sigma-Aldrich Japan, Tokyo, Japan) and immediately cooled on ice.

A portion of the *longissimus dorsi* muscle was collected approximately 15 cm from the rear end of left scapula and immediately cooled on ice. The samples of blood, digestas, mucosae and muscles were brought to our laboratory under cool condition (on ice) within 10 h. Plasma was separated by centrifugation (3,000 × *g*, 10 min, 4°C). Samples were then stored at −80°C (VT-208; Nihon Freezer) until further use.

### 2.5 Lactulose/mannitol test

We used L/M test to evaluate the leaky gut condition in the weaning period. L/M test is the gold standard method to detect intestinal permeability in humans (39); thus, we adopted this method to evaluate the leaky gut condition in pigs without slaughter.

A solution of LM [lactulose (L; 0.5 g/mL, MONILAC powder, Chugai Pharmaceutical, Tokyo, Japan) and mannitol (M; 0.1 g/mL, Nacalai Tesque, Kyoto, Japan)] was prepared.

All pigs had their feed removed in the evening of day 28 and their body weights were measured. Next, the LM solution (1 mL/kg) was administered orogastrically the next morning (administration started at 10:00 am) with a commercial catheter (J Feed Nutrition Catheter 15F 120 cm, JMS, Hiroshima, Japan). After 4 h, blood was collected from the jugular vein of pigs and cooled on ice immediately. Plasma was separated by centrifugation (3,000 × *g*, 10 min, 4°C; CF9RX, HITACHI) and stored at −80°C (VT-208; Nihon Freezer) until further use.

The L and M concentrations were measured by an LC–MS/MS apparatus (Acquity TQD UPLC-MS/MS system; Waters, Milford, MA, USA) at the Kyoto Institute of Nutrition and Pathology (Kyoto, Japan). Briefly, an internal standard solution was prepared by mixing 500 mg/L of lactulose-^13^C_12_ (Sigma-Aldrich Japan) and D-Mannitol-^13^C_6_ (Sigma-Aldrich Japan) with ultrapure water (Fujifilm Wako, Osaka, Japan). Frozen plasma was thawed and 50 μL were transferred to 2-mL screw-cap tubes (Watson, Tokyo, Japan), to which 5 μL of internal standard solution was immediately added. After mixing, 495 μL of acetonitrile (Fujifilm WAKO) was added, and then homogenized at 3,000 r.p.m. for 30 s using a Micro Smash MS-100 (TOMY, Tokyo, Japan) with a 5.5 *φ* stainless steel ball (TOMY). After homogenization, the microtubes were kept 30 min on ice, then the tubes were centrifuged at 15,000 × *g*, for 10 min, at 4°C (Model3520; Kubota, Tokyo, Japan). The supernatants were collected and filtered with commercial filters (PTFE *φ* 4mm/0.2μm; As ONE, Osaka, Japan). The lactulose and mannitol concentrations in the samples were measured using an ultra-pressure liquid chromatography apparatus equipped with a binary solvent manager, an autosampler, and a column heater and tandem mass spectrometry (Acquity TQD UPLC-MS/MS system; Waters). A chromatographic separation was conducted using an Acquity UPLC BEH Amide column (2.1 × 100 mm; particle size 1.7 μm; Waters). The mobile phase, delivered at a flow rate of 0.2 mL/min at 85°C, was a gradient of solution A (acetonitrile: isopropanol (Fujifilm WAKO): ultrapure water = 90: 5: 5) and solution B (acetonitrile: ultrapure water = 80: 20). The gradient was as follows: a flow of 100% solution A for 5 min, followed by a gradual decrease to 0% solution A for 9 min, and maintenance of the 100% solution B for 8 min. The total run time of this method was 22 min. Solutes were detected using the tandem quadrupole mass spectrometer (Waters) with a Z-spray ion interface. The system was controlled using the Waters MassLynx mass spectrometry software (Waters). Ionization was achieved using an electrospray positive ionization mode. The positive ion mode was set as follows: ion source temperature, 150°C; capillary voltage, 2.0 kV; and desolvation temperature, 450°C. The desolvation and cone gas flow were 900 L/h and 50 L/h, respectively. A multiple reaction monitoring (MRM) was conducted. Transitions, m/z values, and cone voltages are shown in the Supplementary Table S2. The injection volume was 5 μL. The concentrations of lactulose and mannitol were calculated from the peak areas detected in the chromatogram with MRM in relation to the respective internal standard.

Prior to this, all pigs were confirmed to be negative in lactulose and mannitol in the blood.

### 2.6 Measurement of blood parameters related to liver functions

Plasma collected on days 0, 14, 28, 42, 56, 70, 84, 98, 112, and 126 had the aspartate aminotransferase (AST) and alanine aminotransferase (ALT) concentrations measured using the Japan society of clinical chemistry transferable method by a commercial laboratory (Fujifilm VET systems, Tokyo, Japan).

### 2.7 Measurement of short-chain fatty acid and lactate concentrations in the digesta and blood

The short-chain fatty acid (SCFA) and lactate concentrations in the cecal digesta were measured by ion-exclusion high pressure liquid chromatography (Shimadzu, Kyoto, Japan) as described elsewhere (40). The SCFA concentrations in plasma of ileal and cecal veins were measured by gas chromatography–mass spectrometry (Shimadzu) as described elsewhere (41).

### 2.8 mRNA expression analyses by real-time PCR

Total RNA was extracted from the mucosae in the same manner as previously described (42) with some modifications. Briefly, total RNA was extracted from the respective portions of mucosae using a QuickGene RNA tissue kit SII (KURABO, Osaka, Japan) and an RNA extraction kit for use with a semi-automated nuclear acid extraction machine (QuickGene Mini-480; KURABO). The concentration of the extracted total RNA was measured with a spectrophotometer (Infinite 200 PRO M Nano; TECAN Japan, Kanagawa, Japan), and 500 ng of the total RNA was used for reverse transcription. Afterwards, reverse transcription was conducted using a commercial kit (PrimeScript RT Reagent Kit, TakaraBio, Shiga, Japan) with oligo (dT)20 and random primers, which were part of the kit. All procedures were conducted according to the manufacturer’s instruction.

Real-time polymerase chain reaction (PCR) was conducted using a Rotor-Gene Q (Qiagen, Tokyo, Japan). Primers (FASMAC, Kanagawa, Japan) and TaqMan probes (Universal ProbeLibrary Set and Human and Extension Set; Roche Applied Science, Penzberg, Germany) used in the present study are listed in the Supplementary Table S3. Target genes were selected from our previous study (23). Optimal primers and probes were designed using freely available online tools.^1^ The relative expression levels of mRNA were calculated by the 2^-*ΔΔCt*^ method (43). *ΔΔCt* value was calculated as follows: *ΔΔCt* = (*Ct*_target gene_ – *Ct*_beta-actin_)_group PP or PC_ – (*Ct*_target gene_ – *Ct*_beta-actin_)_group CC_.

### 2.9 Triglyceride concentration and fatty acid compositions in the muscle

Triglyceride concentration was measured by a previously described method (44) with some modifications. Briefly, the center segment (approximately 5 g) of the *longissimus dorsi* muscle was sub-sampled and chopped with a surgery scissor (As ONE). After mixing, 90 mg of the chopped meat of each sample was transferred to 2-mL screw-cap microtubes (Watson), then 1.710 mL of extractant [chloroform (Guaranteed reagent, cat. 038–02606; Fujifilm WAKO): methanol (for LC/MS, cat. 134–14523; Fujifilm WAKO) = 2: 1] and a 5.5 *φ* stainless steel ball (TOMY) were added. The muscle was homogenized at 3,800 r.p.m. for 120 s with the Micro Smash MS-100 (TOMY). After homogenization, the microtubes were vortexed for 30 min at room temperature, and then 200 μL of ultrapure water (Fujifilm WAKO) was added. The microtubes were vortexed again and then centrifuged at 10,000 × *g*, for 5 min, at 4°C (Model3520; Kubota). The chloroform layers were collected and transferred to new 2-mL microtubes (Watson). The solvent was evaporated using a centrifugal evaporator (CVE-2200, EYELA, Tokyo, Japan) at 60°C. Dried extracts were then suspended in 90 μL of isopropanol (Fujifilm WAKO). The suspension was used to measure the triglyceride concentration using a commercial kit (Triglyceride Test WAKO; Fujifilm WAKO), as per the manufacturer’s instructions.

To measure the fatty acid compositions in the muscles, first, an internal standard solution was prepared by diluting 1 mg/mL of methyl heptadecanate (Sigma-Aldrich Japan) in methanol (Fujifilm Wako). A total 600 mg of the chopped muscle was inserted to 2-mL screw-cap microtubes (Watson), and then 0.3 mL of ultrapure water (Fujifilm Wako) and a 5.5 *φ* stainless steel ball (TOMY) were added. The muscle was minced at 3,800 r.p.m. for 120 s with the Micro Smash MS-100 (TOMY). After mincing, 100 mg of slurry were sub-collected to new 2-mL screw-cap microtubes (Watson), and 50 μL of internal standard solution, 1.6 mL of extractant [chloroform (Fujifilm WAKO): methanol (Fujifilm WAKO) = 2: 1] and a 5.5 *φ* stainless steel ball (TOMY) were added. The sample was homogenized at 3,800 r.p.m. for 120 s with the Micro Smash MS-100 (TOMY). After homogenization, the microtubes were vortexed at 30 min at room temperature, and then 200 μL ultrapure water (Fujifilm WAKO) was added. After further vortexing, the microtubes were then centrifuged at 10,000 × *g*, for 5 min, at 4°C (Model3520; Kubota). The chloroform layer was collected and transferred to new 2-mL microtubes (Watson), and evaporated using the centrifugal evaporator (CVE-2200, EYELA) at 37°C. The dried extracts were suspended in 400 μL of methylation reagent A and 400 μL of methylation reagent B [these reagents were part of the commercial kit named Fatty Acid Methylation Kit (Nacalai Tesque)]. The mixtures were stored at room temperature for 1 h. Next, 400 μL of methylation reagent C was added to the mixtures, vortexed, and stored at room temperature in 20 min. Finally, 400 μL of the isolation reagent was added and vortexed. The upper layer was collected and used for further analysis. Fatty acids such as methyl linoleate, methyl oleate, methyl palmitate and methyl stearate were measured using a gas chromatography apparatus equipped with a flame ionization detector (GC-2014-FID; Shimadzu). The chromatographic separation was conducted using a ZB-FFAP column (ID: 0.32 mm × 15 m; film thickness: 0.25 μm; Shimadzu). Helium (Masuda Medical Instrument, Kyoto, Japan) was used as the carrier gas, and the flow rate was set at 35 cm/s. The thermal conditions of the column oven was 210°C, the injection port temperature was 260°C, the flame ionization detector temperature was 260°C, and the split ratio was 40:1.

### 2.10 Microbiome analysis of the cecal digesta

Bacterial DNA was extracted from the cecal digestas using a commercial extraction kit (QuickGene DNA tissue kit; KURABO) as described elsewhere (45).

Library preparation and deep sequencing by MiSeq (Illumina K.K., Tokyo, Japan) were carried out exactly as described by Inoue et al. (46). Briefly, the V3–4 region of the 16S rRNA genes in each sample was amplified by primers 341F and 805R containing a 5′ overhang adapter sequence for PCR by a KAPA HiFi HotStart Ready Mix (Kapa Biosciences, Wilmington, MA, USA). The amplicon was purified with NucleoFast 96 PCR plates (Takara Bio). A second PCR was carried out using the KAPA HiFi HotStart Ready Mix to attach a unique combination of dual indices (I5 and I7 indices) and Illumina sequencing adapters to each sample. The amplicon of the second PCR was purified and the concentration was normalized using a SequalPrep Normalization Plate Kit (Life Technologies, Tokyo, Japan). Each of the normalized amplicons was then evenly pooled and concentrated using AMPure XP beads (Beckman Coulter, Tokyo, Japan). The quantity of the library was assessed with a Library Quantification Kit for Illumina (Kapa Biosciences). The library was denatured with 0.2N NaOH (SIGMA-Aldrich Japan) and combined with phiX Control (v3, Illumina, San Diego, CA, USA; expected 20%). Eleven picometers of the library, combined with phiX Control, was heat-denatured at 96°C for 2 min and sequenced using a 285 bp paired-end strategy on the Miseq apparatus (Illumina K.K.) as per the manufacturer’s instructions.

After the sequencing, quality filtering, denoising, determination of amplicon sequence variants (ASVs), and taxonomic classification of ASVs against the SILVA 138 database, the obtained reads were analyzed by QIIME2 (ver. 2023.2) with DADA2 plugin. Denoising using DADA2 was conducted with the trimming length from the left set at 17 and that from the right at 19. The truncation length was set at 250 for both reads. Singletons and ASVs assigned to chloroplasts and mitochondria were removed in this study.

### 2.11 Statistical analysis

The daily clinical score in each pen was summed in the weaning, growing and fattening stages.

The parameters of growth performance (DG and FCR; CC group, *n* = 4 and P group, *n* = 8) and blood parameters (L/M ratio, AST and ALT concentration; CC group, *n* = 8 and P group, *n* = 16) in the weaning period (days 0–42) were analyzed to investigate the homoscedasticity by the F test. According to the results, either the Student’s or the Welch’s *t*-test was used to analyze differences between the groups. The summed clinical score in the weaning period was analyzed by the Mann–Whitney test to determine the differences between groups.

The parameters of growth performance (DG and FCR; *n* = 4), blood parameters (AST and ALT concentrations), SCFA concentrations, mRNA expression in mucosae, fatty and amino acid concentrations in muscles, and the microbiome parameters in ceca (CC group, *n* = 7; PP and PC groups, *n* = 8) were analyzed to investigate the homoscedasticity by the Bartlett test. According to the results, either a completely randomized design one-way analysis of variance or the Kruskal–Wallis test was used to analyze the differences between the groups. Either Tukey or Steel-Dwass *post-hoc* comparisons were used for multiple comparisons, as needed. The summed clinical scores in the growing and fattening periods were analyzed by the Kruskal–Wallis test to determine the differences between groups. Steel-Dwass *post-hoc* comparisons were used for multiple comparisons, as needed.

In all the statistical analyses, the values are the arithmetic means ± the standard errors. In addition, the differences between the means were considered significant if *p* < 0.05, and with a tendency to be significant if *p* < 0.1. All statistical analyses were conducted using R software version 4.1.2 (R Core Team, Vienna, Austria).

## 3 Results

### 3.1 Scoring criteria for clinical symptoms and abnormalities at slaughter

Total diarrhea scores during the study were 20.8, 17.3 and 9.8 for the pigs in the groups control, PP and PC, respectively, indicating that there were no statistical differences (*p* = 0.30). Total vitality and appetite scores during the study were 0.3, 0.0, and 0.0 for the pigs in the groups control, PP and PC, respectively, indicating that there were no statistical differences. Total respiratory difficulty scores during the study were 1.0, 0.3, and 3.5 for the pigs in the groups control, PP and PC, respectively, again, indicating that there were no statistical differences. In addition, as mentioned above, a pig died of acute pleuropneumonia on day 90.

At the slaughter day, macroscopic abnormalities of organs were checked; remarkable abnormalities were not found in any of the pigs.

### 3.2 Growth performance parameters

**Figure 2:**
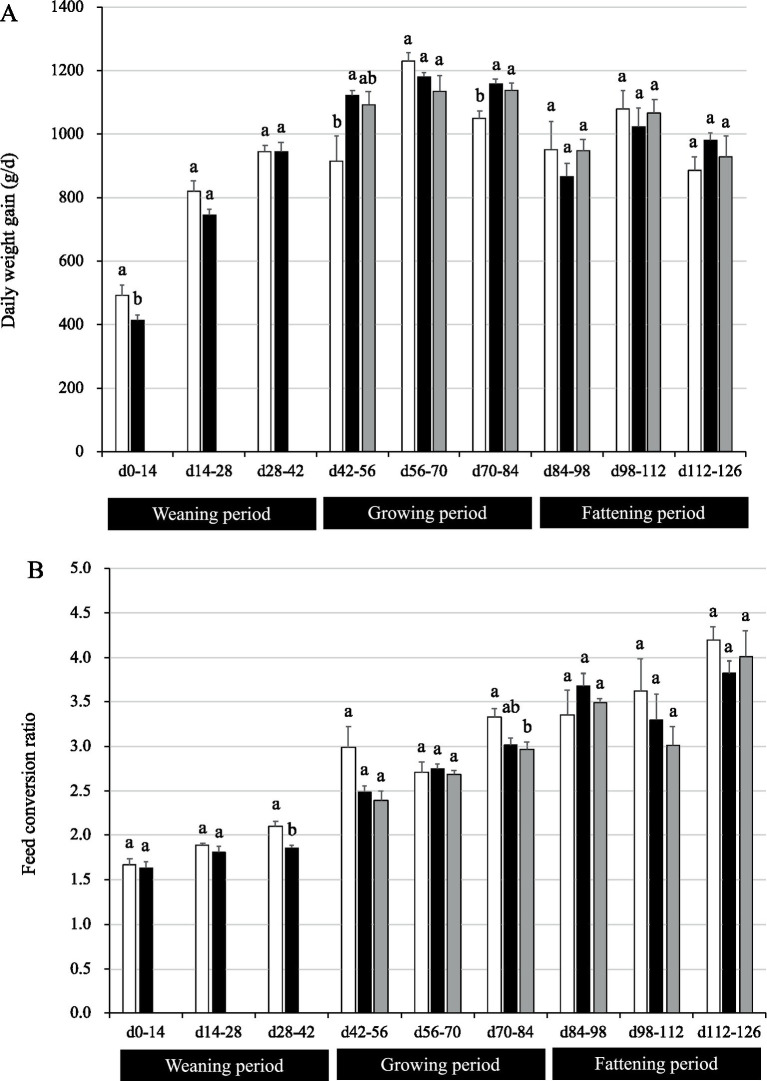
Growth performance of pigs with or without *H. coagulans* SANK70258 administration. Bars indicate the arithmetic means and error bars indicate the standard errors. Open bar, control (CC; *n* = 4); Close bar, pigs supplemented with *H. coagulans* SANK70258 at the weaning (P; *n* = 8) and growing and fattening (PP; *n* = 4) periods. Gray bar, pigs supplemented with *H. coagulans* SANK70258 at the weaning period but not at the growing and fattening periods (PC; *n* = 4). **(A)** Daily weight gain; **(B)** feed conversion ratio. The parameters in weaning period were analyzed to investigate the homoscedasticity by the F test. According to the results, either the Student’s or the Welch’s *t*-test was used to analyze differences between the groups. The parameters in growing and fattening periods were analyzed to investigate the homoscedasticity by the Bartlett test. According to the results, either a completely randomized design one-way analysis of variance or the Kruskal–Wallis test was used to analyze the differences between the groups. Either Tukey or Steel-Dwass *post-hoc* comparisons were used for multiple comparisons, as needed. Different letters indicate significant differences between groups (*p* < 0.05).

During the weaning period, the pigs in the P group had a significantly lower DG than the control pigs during the first 14 days (*p* = 0.04; Figure 2A). The reduction of DG became unclear after day 14; DG tended to be lower in P group rather than in CC group with no statistical significances on days 14–28, and then DG became similar between CC and P groups on days 28–42. During the growing period, pigs in group PP showed a significant increase in DG on days 42–56 (*p* = 0.04). In addition, pigs in groups PP and PC had higher DG on days 70–84 (*p* = 0.01; Figure 2A). Nonetheless, no differences were observed between groups on days 56–70. During the fattening period, no differences were observed between groups on days 84–98, 98–112 and 112–126.

During the weaning period (days 28–42), group P had a lower FCR than control (*p* = 0.004; Figure 2B). Nonetheless, no differences were observed between groups on days 0–14 and 14–28. During the growing period (days 70–84), pigs in group PC had a lower FCR than the CC pigs (*p* = 0.03). Nonetheless, no differences were observed between groups on days 42–56 and 56–70. During the fattening period, no differences were found between groups on days 84–98, 98–112, and 112–126.

### 3.3 Lactulose/mannitol ratio in weaning period

**Figure 3:**
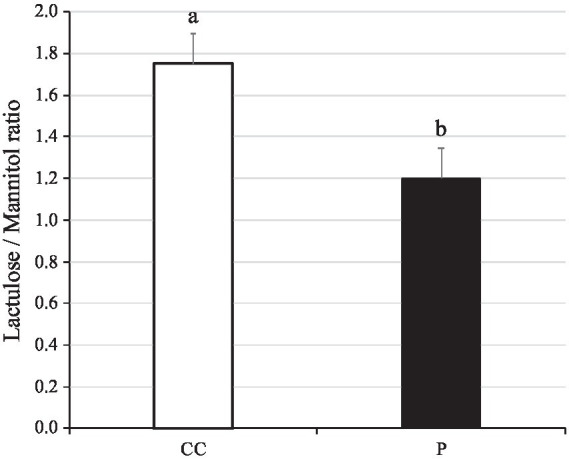
Lactulose/Mannitol ratio in plasma of weaning pigs with or without *H. coagulans* SANK70258 administration. Bars indicate the arithmetic means and error bars indicate the standard errors. Open bar, control (CC; *n* = 8); Close bar, pigs supplemented with *H. coagulans* SANK70258 (P; *n* = 16). The parameter was analyzed to investigate the homoscedasticity by the F test. According to the results, either the Student’s or the Welch’s *t*-test was used to analyze differences between the groups. Different letters indicate significant differences between groups (*p* < 0.05).

The lactulose/mannitol (L/M) ratio is used to assess the integrity of the gut. The L/M ratio in blood plasma was found to be lower in pigs supplemented with SANK70258 than in control (*p* = 0.03; Figure 3). In addition, according to a previous report, L/M ratio in blood after 4 h of L and M inoculation was approximately 0.25–2.5 (39) in weaned pigs, hence the values of our results seemed reasonable.

### 3.4 Aspartate and alanine aminotransferase in plasma

**Figure 4:**
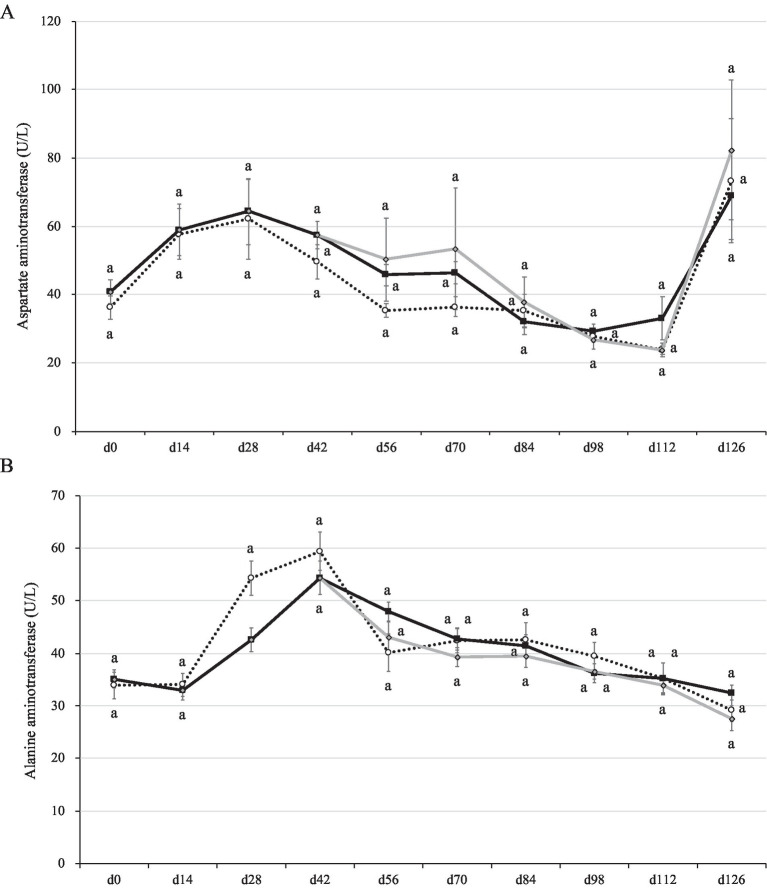
Aspartate and alanine aminotransferase concentrations in the plasma of pigs with or without *H. coagulans* SANK70258 administration. Plots indicate the arithmetic means and error bars indicate the standard errors. Dotted line, control (CC; *n* = 8); Black line, pigs supplemented with *H. coagulans* SANK70258 at the weaning (P; *n* = 16), growing and fattening (PP; *n* = 8) periods; Gray line, pigs supplemented with *H. coagulans* SANK70258 at the weaning period but not at growing and fattening periods (PC; *n* = 8). A control pig died on day 90. Afterwards, the means of CC group was calculated with 7 pigs. **(A)** Aspartate aminotransferase concentration; **(B)** alanine aminotransferase concentrations. The parameters in weaning period were analyzed to investigate the homoscedasticity by the F test. According to the results, either the Student’s or the Welch’s *t*-test was used to analyze differences between the groups. The parameters in growing and fattening periods were analyzed to investigate the homoscedasticity by the Bartlett test. According to the results, either a completely randomized design one-way analysis of variance or the Kruskal–Wallis test was used to analyze the differences between the groups. Either Tukey or Steel-Dwass *post-hoc* comparisons were used for multiple comparisons, as needed. Different letters indicate significant differences between groups (*p* < 0.05).

The ranges of AST concentration were 40–60 U/L in the weaning period, 30–50 U/L in the growing period, and 20–80 U/L in the fattening period (Figure 4A). The ranges of ALT concentration were 30–60 U/L in the weaning period, 40–50 U/L in the growing period, and 30–40 U/L in the fattening period (Figure 4B). In a previous report, the AST concentration was 55–65 U/L in the growing period and 55–70 U/L in the fattening period, whereas the ALT concentration was 20–30 U/L in the growing period and 20–33 U/L in the fattening period (47). Comparing these studies, the parameters were generally similar, but those of the present study were slightly higher than those of the previous report. Hepatitis E virus infection induces the AST secretion into the serum as high as 100 U/L in shipping pigs (48). However, the pigs used in the present study seemed to be within a normal range (not infected) throughout the study except for the day 126 (70–80 U/L in range).

The AST concentration in plasma did not change between groups during this study. However, the ALT concentrations significantly decreased in the blood plasma of pigs in groups P and CC on day 28 (*p* = 0.01; Figure 4B). By day 126 (slaughter day), pigs in all pig groups had drastic increases of AST in blood plasma (Figure 4A).

### 3.5 Fatty acids composition in the longissimus dorsi muscles of fattening pigs

**Figure 5:**
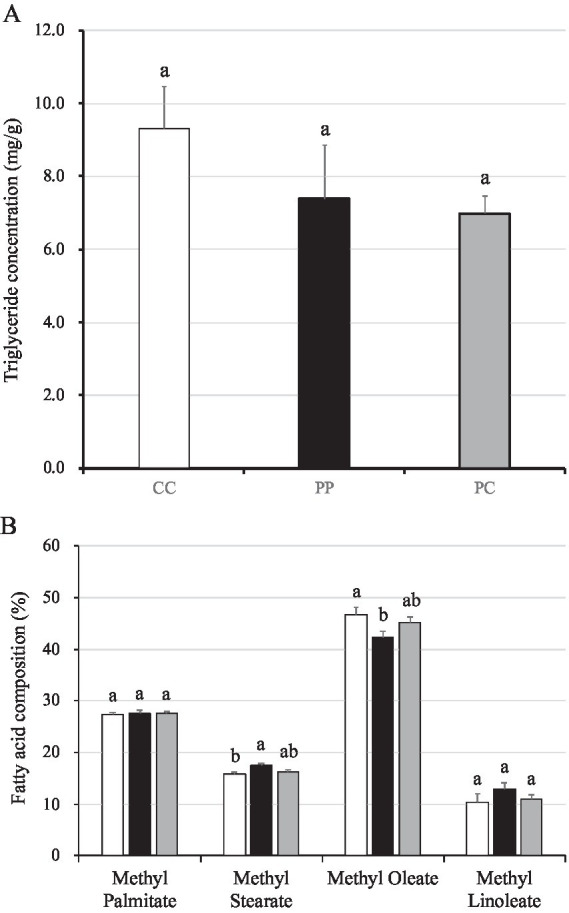
Triglyceride concentration and fatty acid composition in muscle of pigs with or without *H. coagulans* SANK70258 administration. Bars indicate the arithmetic means and error bars indicate the standard errors. Open bar, control (CC; *n* = 7); Close bar, pigs supplemented with *H. coagulans* SANK70258 (PP; *n* = 8). Gray line, pigs supplemented with *H. coagulans* SANK70258 at the weaning period but not at growing and fattening periods (PC; *n* = 8). **(A)** Triglyceride concentration in muscle; **(B)** fatty acid composition in muscle. The parameters were analyzed to investigate the homoscedasticity by the Bartlett test. According to the results, either a completely randomized design one-way analysis of variance or the Kruskal–Wallis test was used to analyze the differences between the groups. Either Tukey or Steel-Dwass *post-hoc* comparisons were used for multiple comparisons, as needed. Different letters indicate significant differences between groups (*p* < 0.05).

There were no differences in the triglyceride concentrations between groups (Figure 5A). However, when compared with pigs in the control group, the percentage of methyl stearate in the fatty acids of PP was higher than in the CC group (*p* = 0.03). In contrast, the percentage of methyl oleate in the fatty acids composition of pigs in group PP was lower than in the CC group (*p* = 0.04; Figure 5B). Nonetheless, no differences were observed in the percentage of methyl palmitate and methyl linoleate between groups.

### 3.6 Short chain fatty acid and lactate concentrations in cecal digesta and ileal and cecal vein

No differences were found in the concentrations of SCFA such as acetate, propionate, *n*-butyrate, *iso* -butyrate, *n*-valerate and *iso*-valerate in the cecal digesta between groups, although the concentration of lactate in cecal digesta was relatively high in pigs in the PP group (*p* = 0.31; Supplementary Figure S1). No differences were found in the SCFA concentrations in the plasma of ileal vein between groups. No differences were found in the acetate, propionate, *n*-butyrate, and *n*-valerate concentrations in the plasma of cecal vein between groups, although the concentrations of *iso*-butyrate and *iso*-valerate were lower in the blood in the cecal vein of pigs in the group PP than those of CC pigs significantly (*p* = 0.01 and 0.02, respectively).

### 3.7 Cecal microbiota of fattening pigs

No differences in the alpha diversity of the Chao 1 and Shannon indices of the cecal microbiota were detected between groups (Supplementary Figure S2).

At the genus level, the abundances of nine genera were significantly different (Supplementary Table S4). The relative abundance of genus *Subdoligranulum* was higher in the cecal digestas of pigs in the group PC than those in the other groups. The relative abundances of genera *Solobacterium, Butyricicoccus*, *Bifidobacterium* and *Lachnospiraceae*_ND3007_group were higher in the cecal digestas of pigs in the group PC than those in CC group. Order Enterobacterales unknown family and family *Pasteurellaceae* unknown genus were higher in pigs in the group PP than in the other groups. In addition, the relative abundance of family *Lachnospiraceae* unknown genus was lower in pigs in groups PP and PC than that in CC group. Uncultured family *Atopobiaceae* was higher in both groups PP and PC than that in CC group. The raw data of microbiome at the genus level is shown in the Supplementary Table S5.

### 3.8 Gene expression in the jejunal, ileal, and cecal mucosae

The gene expression levels in jejunal, ileal, or cecal mucosae of pigs in the present study are shown in Table 1. Gene expressions associated with antimicrobial peptide such as *PR39* and *PMAP23* in jejunal, ileal, and cecal mucosae did not change between groups. While the gene expressions associated with tight junction proteins such as *CLDN1*, *OCLN*, and *ZO1* in ileal and cecal mucosae did not change between groups, *ZO1* expression was more stimulated in the jejunal mucosae of PC pigs than in those of CC pigs. The gene expression associated with the IgA secretion such as *PIGR* in jejunal, ileal, and cecal mucosae did not change between groups. The gene expressions of cytokines such as *IFNG, IL6, IL10, IL17A, IL17F* in jejunal, ileal, and cecal mucosae did not change between groups. Nonetheless, in the jejunal mucosae, *IL22* expression was more stimulated in PC pigs than in that of CC pigs.

**Table 1:** Gene expression levels in jejunal, ileal, and cecal mucosae.

Genes	Intestine	CC	PP	PC	*P*-value
Jejunum	*PR39*	4.05 ± 2.26a	11.43 ± 6.44a	8.90 ± 3.19a	0.80
	*PMAP23*	3.09 ± 1.68a	10.20 ± 5.19a	7.55 ± 2.84a	0.43
	*CLDN1*	1.05 ± 0.14a	1.32 ± 0.27a	1.75 ± 0.17a	0.07
	*OCLN*	1.03 ± 0.09a	1.79 ± 0.33a	1.75 ± 0.39a	0.20
	*ZO1*	1.04 ± 0.11b	1.36 ± 0.33ab	1.63 ± 0.08a	0.04
	*PIGR*	1.87 ± 0.82a	1.76 ± 0.72a	3.28 ± 0.64a	0.27
	*IFNG*	1.02 ± 0.08a	1.03 ± 0.15a	0.80 ± 0.11a	0.32
	*IL6*	0.80 ± 0.65a	0.29 ± 0.15a	0.59 ± 0.39a	0.90
	*IL10*	1.18 ± 0.25a	1.71 ± 0.47a	1.94 ± 0.34a	0.37
	*IL17A*	1.13 ± 0.23a	1.47 ± 0.33a	2.08 ± 0.27a	0.08
	*IL17F*	1.23 ± 0.29a	1.50 ± 0.32a	2.59 ± 0.48a	0.04
	*IL22*	1.10 ± 0.18b	1.52 ± 0.29ab	2.51 ± 0.48a	0.03
Ileum	*PR39*	0.94 ± 0.43a	4.50 ± 1.81a	6.07 ± 3.44a	0.14
	*PMAP23*	2.08 ± 0.86a	3.55 ± 1.00a	4.00 ± 1.71a	0.56
	*CLDN1*	1.29 ± 0.31a	0.91 ± 0.25a	2.56 ± 1.36a	0.73
	*OCLN*	1.14 ± 0.22a	0.82 ± 0.13a	2.01 ± 0.89a	0.43
	*ZO1*	1.03 ± 0.10a	0.84 ± 0.06a	1.14 ± 0.24a	0.34
	*PIGR*	1.50 ± 0.54a	0.96 ± 0.21a	1.82 ± 0.76a	0.86
	*IFNG*	1.25 ± 0.35a	0.78 ± 0.10a	1.08 ± 0.36a	0.77
	*IL6*	1.13 ± 0.50a	3.32 ± 0.79a	4.41 ± 1.42a	0.18
	*IL10*	1.15 ± 0.22a	1.13 ± 0.21a	3.30 ± 1.94a	0.93
	*IL17A*	1.12 ± 0.20a	0.96 ± 0.22a	3.50 ± 2.26a	0.71
	*IL17F*	1.30 ± 0.33a	1.15 ± 0.34a	2.61 ± 1.61a	0.83
	*IL22*	1.21 ± 0.27a	0.86 ± 0.16a	2.94 ± 1.77a	0.70
Cecum	*PR39*	1.67 ± 0.71a	2.28 ± 0.37a	6.93 ± 3.56a	0.55
	*PMAP23*	1.02 ± 0.31a	2.24 ± 0.79a	2.92 ± 1.37a	0.74
	*CLDN1*	1.28 ± 0.32a	0.98 ± 0.27a	1.68 ± 0.51a	0.43
	*OCLN*	1.25 ± 0.29a	1.20 ± 0.21a	3.19 ± 1.07a	0.53
	*ZO1*	1.14 ± 0.27a	0.73 ± 0.15a	0.75 ± 0.08a	0.10
	*PIGR*	1.16 ± 0.28a	1.07 ± 0.21a	0.83 ± 0.22a	0.60
	*IFNG*	1.12 ± 0.22a	1.23 ± 0.17a	1.79 ± 0.50a	0.60
	*IL6*	2.66 ± 1.54a	1.96 ± 1.12a	0.61 ± 0.33a	0.36
	*IL10*	1.56 ± 0.54a	1.10 ± 0.29a	2.17 ± 0.77a	0.41
	*IL17A*	1.33 ± 0.40a	0.96 ± 0.21a	2.07 ± 0.73a	0.74
	*IL17F*	1.31 ± 0.37a	0.98 ± 0.31a	2.08 ± 0.69a	0.28
	*IL22*	1.45 ± 0.47a	0.96 ± 0.21a	1.60 ± 0.48a	0.50

Different letters indicate significant differences between groups (*P* < 0.05). The parameters were analyzed to investigate the homoscedasticity by the Bartlett test. According to the results, either a completely randomized design one-way analysis of variance or the Kruskal–Wallis test was used to analyze the differences between the groups. Either Tukey or Steel-Dwass *post-hoc* comparisons were used for multiple comparisons, as needed.

## 4 Discussion

The key mechanism of *H. coagulans* SANK70258 was “anti-inflammatory” and/or “suppression of inflammation” (23, 27). Previously, the administration of *H. coagulans* SANK70258 to broilers was shown to improve the growth performance of broilers infected with coccidiosis (21). Administering SANK70258 to broilers improved the immune (gene expressions of *IL10* and *TGFb*) and the barrier (gene expressions associated with tight junction protein) functions in the duodenal mucosa (23). We hypothesized that, similarly in pigs, the improvement of these functions could contribute to enhance the growth performance of pigs. Therefore, in the present study, we evaluated the growth performance of pigs with changes in the intestinal immune and barrier functions induced by SANK70258 administration.

In the present work, supplementation with SANK70258 during the weaning period did not significantly increase growth performance; on the contrary, for the first 14 days, pigs in group P had a lower DG than control pigs. Usually, many pathogens infect commercial pigs during the weaning period. Therefore, weaned pigs are afflicted with a variety of diseases, not only gastrointestinal conditions such as colibacillosis, coccidiosis, and rotavirus infection (33), but also systemic diseases such as the porcine respiratory and reproductive syndrome (PRRS) and the porcine circovirus type 2 infection (49). To prevent pathogen infections, the acute inflammatory response is effective (35). For example, the inflammatory stimulation suppresses the PRRS virus replication in piglets (50). In our previous study, a postbiotic administration enhanced the inflammatory response and prevented colibacillosis in weaning pigs (51). On the other hand, in our previous work, SANK70258 was categorized as the anti-inflammation-inducing probiotic (23, 27). Suppression of the inflammatory response in enterotoxigenic *E. coli* challenged weaned pigs was associated with increased intestinal injury and clinical disease (52). We hypothesized that suppression of the inflammatory response induced by SANK70258 administration was also sensitive to the pathogens and/or harmful materials. Reduction of DG on days 0–14 by SANK70258 administration may have been caused by the same reason. Indeed, in the present study, although the administration of SANK70258 suppressed DG from day 0–14 (Figure 2A), the leaky gut condition improved during the weaning period (Figure 3). This effect (leaky gut improvement) was also observed in our previous broiler study (23). It is well known that weaning stress induces the leaky gut condition and this gut dysfunction causes disease susceptibility (53). An improvement of the leaky gut condition also caused an improvement in the FCR during days 28–42 in group P (Figure 2B). Leaky gut induces harmful materials translation such as pathogenic bacteria, toxins and harmful antigens (5, 54). These materials may affect the growth performance of pigs because these factors induce inflammation (55). Therefore, the outcome of the administration of SANK70258 is not always an improved growth performance but instead, an improvement of the leaky gut condition in weaning pigs.

During the growing period, supplementation with SANK70258 increased significantly the DG of pigs in PP group (Figure 2A). Previous work reported that increasing the supplementation of probiotics to pigs during the growing and fattening periods significantly improved DG (56). Pigs are less likely to be infected by pathogens during the growing period than during the weaning period (33). Furthermore, chronic inflammation, caused by stress and inflammation-inducing ingredients in feed, is always found in intensive livestock production systems; thus anti-inflammation-inducing materials are beneficial to promote growth performance in animals (35). In the present work the administration of SANK70258 stimulated an anti-inflammatory response and/or suppressed inflammatory responses (23, 27, 57), which were later reflected in the growth-promoting effect observed during the growing period because of the chronic inflammation downregulation (Figure 2A).

It has been previously shown that supplementation with probiotic *Clostridium butyricum* helped lower the ALT concentration in sera of growing pigs (58). In addition, Wang et al. (59) reported that supplementation with *Clostridium butyricum* and *Enterococcus faecalis* to weaned pigs lowered the levels of AST and ALT in the sera of weaned piglets. In the present study, there were no differences in the AST level between groups, although all pigs experienced a drastic increase by day 126. The cause of this increase remains unclear, but it can be speculated that pigs were infected with a pathogen by the end of the fattening period, because our previous study suggested that the pigs in the fattening period are likely to be infected by pathogens again in commercial swine farms (33). Furthermore, in the present study, a pig in CC group died of acute pleuropneumonia during the fattening period, which seems to supports our speculation. Although the infection was not substantial enough to make all animals in the present study symptomatically sick, acute inflammatory conditions were likely present in the fattening period, particularly on day 126.

Whereas AST is diffusely represented in the heart, skeletal muscle, kidneys, brain, and red blood cells, ALT has low concentrations in skeletal muscle and kidney. Therefore, an increase in ALT serum levels is more specific associated with liver damage (60). In the present work, the administration of SANK70258 helped lower the ALT concentration of weaned pigs in the P group on day 28 (Figure 3B). In our previous study with humans, consecutive 8-week SANK70258 administration induced a reduction of ALT but not AST (61) in the blood. It can be speculated that only the ALT reduction was the characteristic of this probiotic not only in humans but also in pigs. These results seemed to suggest that supplementation of SANK70258 improved the liver function of pigs, as injury of hepatocytes increases the level of ALT in the blood (62). Many strains of probiotic bacteria have already been applied to the non-alcoholic fatty liver disease and non-alcoholic steatohepatitis patients with AST and/or ALT reduction (18). An improving mechanism of liver dysfunction by SANK70258 was suggested as suppressing the leaky gut (12). It is well known that the leaky gut induces an inflammatory response in the liver (63), and the hepatic inflammation induces a higher ALT concentration in serum (64). SANK70258 was previously reported as an anti-inflammatory probiotic (23, 27, 57), thus, it can be hypothesized that an improved liver function, as suggested by the decreased ALT levels in SANK70258-administered pigs on day 28, may have also meant an improved leaky gut. Another hypothesis of the improving mechanism was the stimulation of anti-oxidant parameters such as super oxide disumutase (SOD) in the liver. A different strain of *H. coagulans* stimulated the SOD activity in the small intestine (65) and liver (66) with improvement of the morphology and function of liver (66). Therefore, SANK70258 may also stimulate the anti-oxidant parameters. Nonetheless, since we did not measure the anti-oxidant, inflammatory and anti-inflammatory parameters in the blood and liver in the present study, this point needs further consideration.

The concentrations of stearate and oleate were higher and lower, respectively, in the *longissimus dorsi* muscles of pigs in the PP group than those of pigs in the PC and control groups (Figure 5). A higher concentration of stearate in pork meat can lower the serum cholesterol level (67). Moreover, oleate is also functional against the blood pressure (68). In the present work, while it remains unclear the reason a longer administration of SANK70258 modified the fatty acid composition in pork meat, it has been previously reported that the administration of certain probiotic bacteria such as *Lactobacillus johnsonii* increases the stearate levels in the fatty acid composition, with an upregulation of several lipid-related genes, including DGAT1, DGAT2, CD36, and PPARγ in the muscle (69). The effect of the administration of SANK70258 on the fatty acid lipid composition of pigs needs further elucidation.

The gene expressions of ZO1 and IL22 were higher in the jejuna of pigs in the group PC than in those in the control and PP groups (Table 1). Protein ZO1 is crucial for mucosal repair (70). IL22 is a member of the interleukin-10 family of cytokines (71). In addition, IL22 is involved in the innate host defense mechanisms against pathogens and plays a role in the protection of the barrier integrity by promoting repair, among other functions, of several tissues and organs (72). These parameters did not change in the pigs in group PP group. We can only speculate that this phenomenon may have been caused by a certain pathogen infection on day 126. For example, according to the microbiome analysis, significant increases in *Pasteurellaceae* and Enterobacterales were observed in group PP (Supplementary Table S4). These bacterial groups encompass potential pathogens. Indeed, *Pasteurellaceae* include genera *Mannheimia* and *Pasteurella* (73), and Enterobacterales include genera *Escherichia*, *Klebsiella*, *Salmonella*, and *Yersinia* (74). Our possible theoretical scenario is as follows. In the group PP, a pathogenic infection occurred in pigs, because the immune response was delayed by the continuous production of anti-inflammatory cytokines, which was caused by the administration of SANK70258. In contrast, in the bodies of pigs in groups CC and PC (pigs withdrawn at weaning from a continuous anti-inflammation response, which would have been induced by the administration of SANK70258), there was a faster immune response, which is the natural response under normal circumstances, and pathogens were quickly eliminated upon infection. Therefore, in the present work, there might have been confounding expressions of mucosal cytokines and the luminal microbiota in the fattening pigs of group PP, which was not observed in the other groups. Nonetheless, to fully elucidate this speculation, further investigation is recommended.

Some limitations were observed in this study. First, we designed a relatively small scale experiment (*n* = 8) to evaluate growth performance. Second, the growth performance of PP pigs in the fattening period became confounding with the induction of liver dysfunction. Third, only single analyses of lactulose/mannitol test were conducted on day 28 in the present study. Although strict effectiveness against leaky gut was evaluated, a pre-trial should be required to understand the internal leaky gut condition of individual pigs. Due to the above limitations, a study using SANK70258 to treat pigs from the weaning to the growing period with a sufficient scale is needed to fully understand the growth promoting effect of SANK70258 in the pigs.

## 5 Conclusion

The consecutive administration of SANK70258 improved the growth performance during the growing period. Pigs supplemented with SANK70258 only during the weaning period had also greater DG and lower FCR toward the end of the growing period. SANK70258-administered pigs experienced an improved liver function (as per the lower ALT levels observed) and hence, a putative improved leaky gut condition in the weaning period. As a result, these pigs also experienced an enhanced growth performance during the growing period.

Since SANK70258 is known as an anti-inflammation-inducing probiotic strain, we hypothesized that the promoting effect of growth performance by SANK70258 depended on the inflammation status of pigs. Weaned pigs are usually infected with many pathogens, therefore an acute inflammation response is required to prevent pathogen infections. The SANK70258 administration was not adequate for pigs during the acute-inflammation phase because SANK70258 suppressed the acute-inflammatory response. A consecutive SANK70258 administration during the growing period, a low risk stage of pathogen infection, resulted in a sustained anti-inflammatory response in the intestine, which may have been an additional positive effect for pigs in group PP. Nonetheless, during the fattening period, the positive effect of consecutive SANK70258 may have been confounded with the risk of pathogen infection again. To elucidate this confounding result during the growing period, it is recommended that further investigation be carried out in the future.

## Data Availability

The sequence data have been deposited in the NCBI Sequence Read Archive (SRA) under accession number PRJNA1193228. The original contributions presented in the study are included in the article/Supplementary material. Further inquiries can be directed to the corresponding author.

## Footnotes

^1^ https://primers.neoformit.com/
