# Supplementary material for: Heyndrickxia coagulans SANK70258 supplementation improves growth performance, gut health, and liver function in growing pigs
Source: Front Vet Sci. 2025 May 27;12:1537913. doi: 10.3389/fvets.2025.1537913 (PMC12150804; doi:10.3389/fvets.2025.1537913)
Supplement: Supplementary file 2 [file Presentation_1.PPTX]

## Slide 1
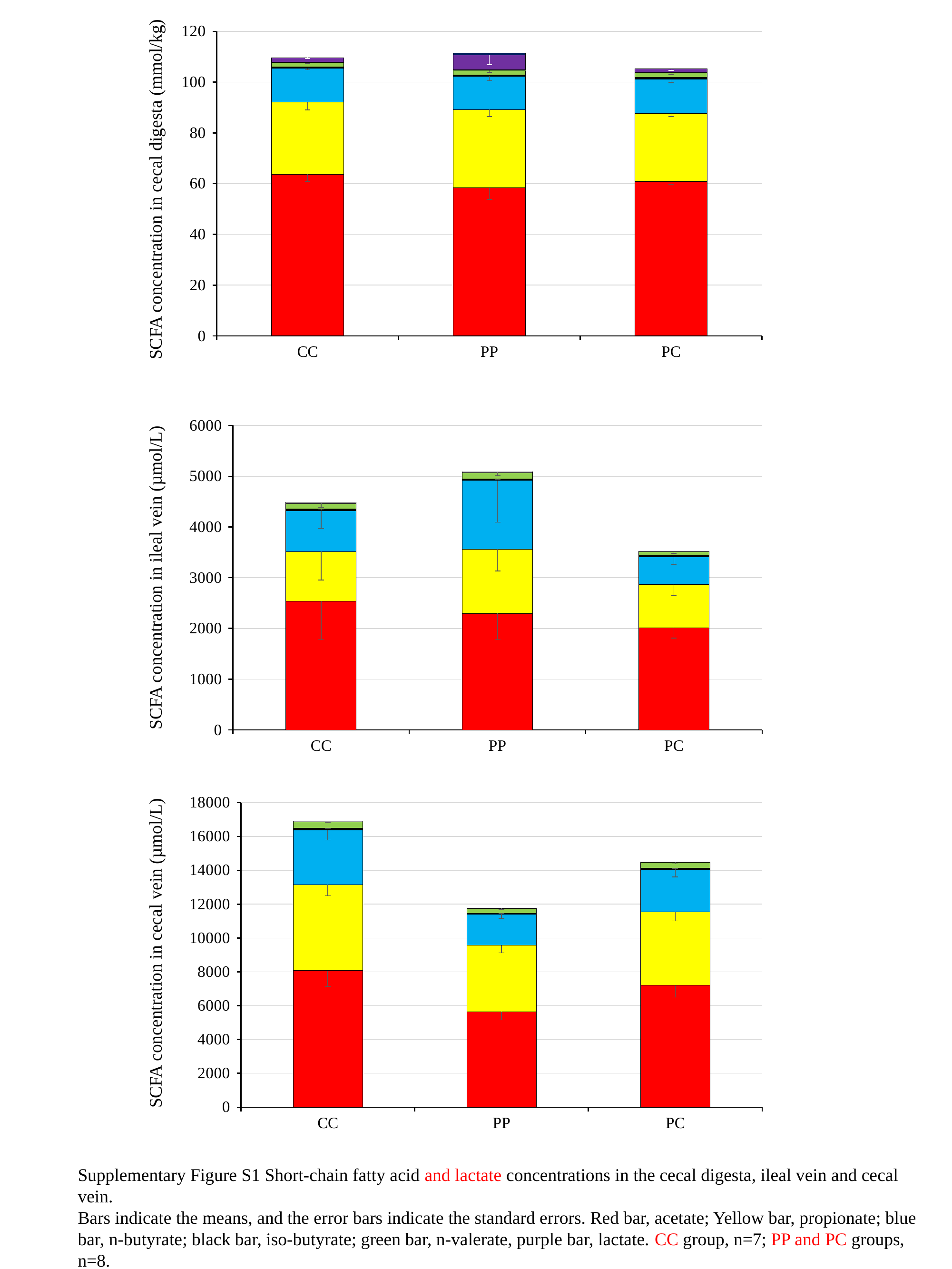

### Chart
| Category | Acetate | Propionate | nButyrate | isoButyrate | nValerate | isoValerate | Succinate | Lactate | Formate |
|---|---|---|---|---|---|---|---|---|---|
| CC | 63.662954861128306 | 28.450395470445038 | 13.245499746850133 | 0.7313387334207622 | 1.3733891995800502 | 0.24942934421489463 | 0.24677866112681993 | 1.6866586914325323 | 0.0 |
| PP | 58.36255198074569 | 30.858731268549427 | 13.12376373167475 | 0.4922015921599111 | 1.7053645952060907 | 0.1340988047054232 | 0.1678453509933082 | 5.802016753827549 | 0.7978222055411324 |
| PC | 60.74251066192437 | 26.968056439525007 | 13.48499322084895 | 0.6150521440847007 | 1.7299204264590482 | 0.12116266284977349 | 0.15393885503982774 | 1.4434362156103415 | 0.0 |SCFA concentration in cecal digesta (mmol/kg)
### Chart
| Category | Acetate | Propionate | nButyrate | isoButyrate | nValerate | isoValerate | Capronate |
|---|---|---|---|---|---|---|---|
| CC | 2539.2269414636844 | 976.1792008083473 | 804.218977216138 | 34.292247431026446 | 108.87398318773754 | 32.2301185349155 | 4.276071919302067 |
| PP | 2294.3644795526798 | 1266.1275177161426 | 1357.6994582161715 | 29.679730705186273 | 125.28592431480817 | 24.557975658190585 | 12.22966385486427 |
| PC | 2013.0647673896572 | 856.5266810925822 | 542.8691102553671 | 24.989784590260623 | 73.98258943405148 | 19.122215556359397 | 7.222410515753036 |SCFA concentration in ileal vein (µmol/L)
### Chart
| Category | Acetate | Propionate | nButyrate | isoButyrate | nValerate | isoValerate | Capronate |
|---|---|---|---|---|---|---|---|
| CC | 8081.113265462074 | 5075.337057827178 | 3213.3934269644765 | 112.22288904728498 | 387.87941557733717 | 79.03339220378435 | 9.212175156431126 |
| PP | 5637.301723700913 | 3925.8842510986633 | 1843.4102538120371 | 54.35120545018975 | 289.32259341422605 | 41.35833016465276 | 10.002525716149787 |
| PC | 7205.376485670723 | 4339.4891414397625 | 2507.8480542319394 | 69.87159995569242 | 347.1932537422009 | 50.49233254612196 | 12.719679256187288 |SCFA concentration in cecal vein (µmol/L)
Supplementary Figure S1 Short-chain fatty acid and lactate concentrations in the cecal digesta, ileal vein and cecal vein.
Bars indicate the means, and the error bars indicate the standard errors. Red bar, acetate; Yellow bar, propionate; blue bar, n-butyrate; black bar, iso-butyrate; green bar, n-valerate, purple bar, lactate. CC group, n=7; PP and PC groups, n=8.

## Slide 2
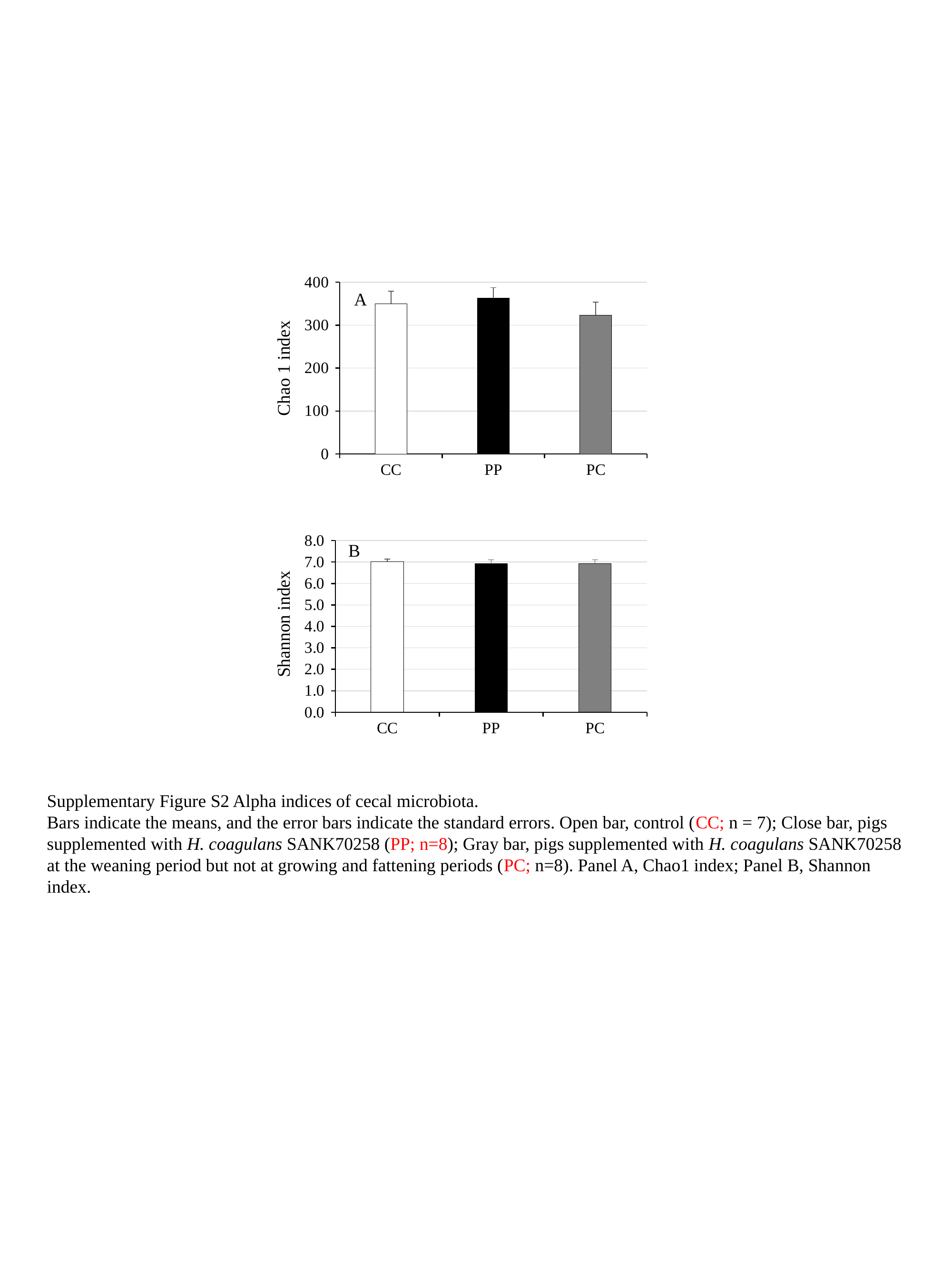

### Chart
| Category | chao1 |
|---|---|
| CC | 349.76972789999996 |
| PP | 362.49702379999997 |
| PC | 323.447916675 |A
Chao 1 index
### Chart
| Category | shannon |
|---|---|
| CC | 7.015582628714286 |
| PP | 6.9240331725 |
| PC | 6.922355846874999 |B
Shannon index
Supplementary Figure S2 Alpha indices of cecal microbiota.
Bars indicate the means, and the error bars indicate the standard errors. Open bar, control (CC; n = 7); Close bar, pigs supplemented with H. coagulans SANK70258 (PP; n=8); Gray bar, pigs supplemented with H. coagulans SANK70258 at the weaning period but not at growing and fattening periods (PC; n=8). Panel A, Chao1 index; Panel B, Shannon index.
